# Gene Expression and Immunochemistry Analysis of ADAMTS-1 and Versican in Ameloblastoma

**DOI:** 10.1155/2022/5235376

**Published:** 2022-10-26

**Authors:** Osvaldo Rodrigues de Souza Neto, Hellen Thais Fuzii, Suély Vieira Da Silva, Vanessa Morais Freitas, João de Jesus Viana Pinheiro

**Affiliations:** ^1^Cell Culture Laboratory, School of Dentistry, Federal University of Para, Rua Augusto Correa, 01 Guama, 66075110 Belem, PA, Brazil; ^2^Immunopathology Laboratory, Tropical Medicine Institute, Federal University of Pará, Av Generalíssimo Deodoro, 92 Umarizal, Belém, PA, Brazil; ^3^Department of Cell and Developmental Biology, Institute of Biomedical Sciences, University of São Paulo, São Paulo, Brazil

## Abstract

**Background:**

Ameloblastoma is a benign but locally invasive odontogenic epithelial tumor, associated with a high recurrence rate after treatment. The action of enzymes of the metalloproteinase family is important to the degraded extracellular matrix, contributing to invasion. Thus, this study aimed to investigate the gene and protein expression of ADAMTS-1 and versican in ameloblastoma.

**Materials and Methods:**

Twenty cases of ameloblastoma (*n* = 20) and ten dental follicles (DF) (*n* = 10) were used as a source for immunochemistry and quantitative RT-PCR for determining the protein and mRNA expressions of the concerned genes, respectively. Moreover, western blot and indirect immunofluorescence analysis were performed in AME cells.

**Results:**

ADAMTS-1 and versican were overexpressed in DF than ameloblastoma by RT-PCR. However, in the immunolocalization analysis, ADAMTS-1 was expressed in ameloblastoma more than in DF and versican immunostaining obtained a similar pattern between ameloblastoma and DF. Indirect immunofluorescence detected the ADAMTS-1 and versican expression in cell lines derived from ameloblastoma. Western blot from cell lysate and conditioned medium detected ADAMTS-1 bands representing full-length and different processed forms. Monensin treatment confined ADAMTS-1 in the cell cytoplasm. Versican fragments also were detected in different compartments, intracellular and conditioned medium, allowing the versican process by ADAMTS-1.

**Conclusion:**

This study showed a distinct expression of ADAMTS-1 and versican in ameloblastoma and DF, with ADAMTS-1 protein higher expression observed in ameloblastoma and possibly cleaved versican. These findings suggested that ADAMTS-1 may participate in tumor invasion, especially for the degradation of substrates (versican) in the ECM.

## 1. Introduction

Odontogenic tumors (OTs) constitute a group of lesions, neoplastic, affecting the maxillofacial region, which may present heterogeneous behavior with histopathological features and various clinical manifestations [1]. Such lesions can be originating from epithelial, mesenchymal, or ectomesenchymal tissues, derivatized from potential cells able to induce the formation of dental tissue and their attachment [2].

Ameloblastoma is a benign tumor, locally invasive, originated from an odontogenic epithelium with variable pathological clinical appearance, high relapse, and eventually undergoes malignant transformation [3, 4].

The tumors are typically surrounded by a connective tissue that forms the stroma, consisting of cells, including fibroblasts and myofibroblasts, arranged in a complex and organized network of supported macromolecules, known as the extracellular matrix (ECM). In the tumor microenvironment, the role of the ECM is not limited only to acting as a barrier to tumor invasion but also works as a reservoir for ligand proteins and growth factors that influence the behavior of tumor cells [5–8]. The cell invasion is related to the action of enzymes of the metalloproteinases family [9–11], such as ADAMTS [12].

A disintegrin and metalloproteinase with thrombospondin motifs (ADAMTS) includes a family of zinc-dependent endopeptidases that are capable of degrading components of the ECM and basal layer, participating in physiologic events and pathologic processes and facilitating growth, invasion, and tumor metastasis [13, 14].

ADAMTS proteases exhibit a common multidomain structure. The backbone organization consists of a prodomain, a catalytic motif, and a disintegrin-like module, linked to an additional *C*-terminal sequence, referred to them as an ancillary domain. This region includes at least one thrombospondin (TSR), a cysteine-rich domain, and a spacer fragment that may or may not be followed by a variable number of additional TSR domains and other motifs (CUB, Gon1-like, mucin-like, and lacunin) [13, 15, 16]. The composition of this *C*-terminal region offers a distinctive feature to each member of the family and provides cues as to their potential functional capabilities, binding and anchoring properties, substrate recognition, half-life, and evolutionary trajectory [17, 18].

ADAMTS-1 has specific substrates that include modular proteoglycans such as versican [19–21]. This proteoglycan is a component of ECM and the first obstacle to causing cell invasion and tumor metastasis [7]. In addition, ADAMTS are proteins that exhibit characteristics structural which give them great potential to perform multiple functions, such as cell proliferation, adhesion, invasion, and cell signaling [14, 22].

This study evaluated the ADAMTS-1 expression and its substrate (versican), which are overexpressed in many tumors. These proteins may produce byproducts that signal for the recruitment of growth factors through their tumor, providing a supportive stroma for interaction with tumor parenchyma promoting their growth and invasion [12].

To the best of our knowledge, there has been no approach reported that involves ameloblastoma and ADAMTS-1. In this pilot study, we hypothesized that ameloblastoma may express ADAMTS-1, which can contribute to locally invasive tumor, through the degradation of versican.

## 2. Methods

### 2.1. Sample Selection

Twenty cases of ameloblastoma were retrieved from the files of the department of Oral Pathology of the School of Dentistry of the University Center of Pará (CESUPA, Belém-PA, Brazil). Samples were classified in accordance with their histological patterns, which are eleven follicular, seven plexiform, and two acanthomatous. Ten cases of dental follicle (DF) were included as controls. This study was approved by the Ethics Committee of the Institute of Oncology Research Center of the Federal University of Pará (n°2.371.646).

### 2.2. Immunohistochemistry

Formalin-fixed, paraffin-embedded tissues were studied by immunohistochemistry. Five-micron sections were obtained and mounted on poly-D-lysine-coated slides (Sigma Chemical Corp., St Louis, MO, USA). Sections were dewaxed in xylene and rehydrated in graded ethanol. Antigen retrieval was carried out in the Pascal chamber (Dako, Carpinteria, CA, USA) for 30 seconds. Sections were immersed in 3% H_2_O_2_ in methanol for 20 minutes for the inhibition of endogenous peroxidase activity and then blocked with 1% bovine serum albumin (BSA, Sigma®) in phosphate-buffered saline (PBS) for 1 hour. The slides were incubated with primary antibodies anti-ADAMTS-1 (1 : 50, Abcam, Inc., Cambridge, MA, USA) and anti-versican (1 : 50, Sigma Chemical Corp, St Louis MO, USA). All primary antibodies were diluted in antibody diluent solution (Dako®) and incubated for 1 hour at room temperature. Subsequently, sections were incubated for 30 minutes with EnVision Plus detection system (Dako®). Diaminobenzidine (Sigma®) was used as a chromogen, and the sections were counterstained with Mayer's hematoxylin (Sigma®) and mounted with Permount (Fisher Scientific, Fair Lawn, NJ, EUA).

### 2.3. Immunostaining Evaluation

Immunohistochemical evaluation was performed by measuring the area (*μ*m) and marking a fraction (%) of ADAMTS-1 and versican proteins in ameloblastoma and dental follicles. Brightfield images from five randomly selected images from each sample were acquired using an Axioskop 40 microscope (Carl Zeiss, Germany) equipped with a CCD color camera (AxiocCam MRc, Carl Zeiss). All images were acquired at the same magnification (40x). Areas of diaminobenzidine staining were separated and segmented using the “deconvolution color plug-in” (Gabriel Landini, https://www.dentistry.bham.ac.uk/landinig/software/software.html) from ImageJ software (public domain software, NIMH, NIH, Bethesda, MD, USA, https://rsbweb.nih.gov/ij/). After image segmentation, the area and total color fraction were measured. Differences in the percentages of stained areas in ameloblastoma and DF were quantified. Image acquisition and measurement of diaminobenzidine staining were blinded before quantification by the examiner.

### 2.4. RNA Extraction

Total RNA was extracted from Formalin fixation paraffin embedding (FFPE) samples, using ReliaPrepTM FFPE total RNA Miniprep System kit, according to the manufacturer's protocol (Promega, Madison, WI, USA). The isolated RNA was stored at −80°C until further use.

### 2.5. Measuring the Quality and Quantity of Extracted RNA

RNA was quantified using the Invitrogen Qubit® Fluorometer equipment and Q32852 Quant-iT RNA Assay Kit (Invitrogen, Carlsbad, CA, USA), 100 assays ^*∗*^5–100 ng^*∗*^ (250 pg/uL–100 ng/uL) for samples reading and following the instructions of the manufacturer. Then, the integrity of the samples was analyzed by Agilent 2100 Bioanalyzer equipment, the RNA 6000 Agilent Pico Kit (Agilent, Santa Clara, CA, USA), following the manufacturer's instructions.

### 2.6. Complementary DNA (cDNA) Synthesis

The reverse transcription of samples was performed to obtain the cDNA used in the real-time polymerase chain reaction (RT-PCR). This procedure was performed using the Superscript III Kit (Superscript ® III Reverse Transcriptase—Invitrogen) according to the manufacturer's protocol.

### 2.7. RT-PCR

Relative quantification was performed. For the detection of amplicons, the fluorescent agent SYBR Green was used. Samples were made in duplicate. The oligonucleotide primers used for qPCR reactions were ADAMTS-1 (F: 5′- TGTAGCCCAGATTCCACCTC-3′, R: 5′- CCCCGCAAACACCACATTTA-3′), VERSICAN (F: 5′- CCCCTGTTGTAGAAAATGCCA-3′, R: 5′- TCCATTTCCTAAGCACCGGA-3′), GAPDH (Glyceraldehyde-3-phosphate dehydrogenase) (F: 5′-TCGGAGTCAACGGATTTGG-3′, R 5′-GATGGCAACAATATCCACTTTACC-3′), and *β*-actin (F: 5′-TAATGTCACGCACGATTTCCC-3′, R 5′-TCACCGAGCCCGGCT-3′)

The qPCR reaction was performed using StepOne Plus (Real Time PCR Systems—Applied Biosystems) with SYBR Green reagent (Applied Biosystems). For the reverse transcription reaction, cDNA, SYBR Green PCR master mix (2x), forward and reverse primers (18 *μ*M), and 20 *μ*L autoclaved Milli-Q qsp water were used. After 10 minutes at 50°C for enzyme activation and denaturation for 5 minutes at 95°C, 45 cycles of 95°C for 30 seconds and 60°C for 1 minute were performed. In the end, the thermal dissociation protocol was performed to control the specificity of the reaction.

Results were analyzed by the StepOne™ Software v2.0. For relative quantification, the following calculation was performed: initially, the cycle threshold (CT) was determined, given by the number of cycles in which the fluorescence signal reached the threshold line, the line in which the emission of fluorescence is above the background noise. The CT is invariably in the region corresponding to the exponential phase of amplification, which makes the estimate of the quantification of the transcripts in the original sample more accurate. The CT values of the genes of interest were normalized in relation to the CT of the constitutive gene, GAPDH (*Glyceraldehyde-3-phosphate dehydrogenase*) *β*-actin, resulting in ΔCT, which CT_gene_—CT_constitutive_. Finally, 2^−ΔCT^ was calculated, which is the value that worked as a representative of the relative expression for each gene [23].

### 2.8. Cell Line

The cell line AME-HPV2 was cultured in Dulbecco's Modified Eagle's Medium-F12 (DMEM-F12, Sigma Chemical Co, St. Louis, MO, USA) supplemented with 10% fetal bovine serum (FBS; Cultilab, Campinas, SP, Brazil). Cells were maintained in 75 cm^2^ flasks in a humidified atmosphere of 5% CO_2_ at 37°C [24].

### 2.9. Western Blot

Cells were lysed with RIPA buffer (150 mM NaCl, 1.0% NP-40, 0.5% deoxycholate, 0.1% SDS, 50 mM Tris pH 8.0) containing protease inhibitors (Sigma). After centrifugation (10.000 × *g*) for 10 minutes at 4°C, the supernatants were recovered and quantified (BCA Kit, Pierce Inc, Rockford, IL, USA). Protein from the conditioned medium (1 mL) was obtained by ethanol precipitation. Samples were resuspended in Laemmli buffer containing 62.5 mM Tris-HCl pH 6.8, 2% sodium dodecyl sulphate (SDS), 10% glycerol, 5% mercaptoethanol, and 0.001% bromophenol blue. A total of 30 *μ*g of cellular proteins were electrophoresed on a 10% polyacrylamide gel, transferred to a Hybond ECL nitrocellulose membrane (Amersham), and blocked in Tris-buffered saline buffer (1X TBS) with 5% nonfat milk overnight at 4°C. Following one wash in TBS with 0.05% Tween 20 (TBST), the membranes were probed with antibodies against ADAMTS-1 (1 : 1000, Millipore MAB 1810), versican (1 : 1000, Abcam ab19345), and *β*-actin (1 : 2000, Sigma). The Clarity Western ECL substrate (Bio-Rad) was used to detect proteins on the membrane according to the manufacturer's protocol.

### 2.10. Golgi Complex Disruption

The Golgi network is the major site of proADAMTS-1 processing [25]. Monensin is a known inhibitor of post-Golgi transport [26]. Therefore, to prevent ADMTS-1 secretion and identify it, the cell line AME-HPV2 was treated with Monensin (Sigma) at a concentration of 2 *μ*M. After 24 h, cellular lysate was performed.

### 2.11. Indirect Immunofluorescence

To verify the expression of ADAMTS-1 and versican, cells were cultured on glass coverslips in 24-well plates. Undergoing a process that followed the following steps: fixation in 2% paraformaldehyde for 10 minutes; washing in PBS (Phosphate Buffered Saline); membrane permeabilization with 0.5% Triton X-100 (Sigma) solution for 15 minutes; PBS wash; incubation with 1% PBS/BSA (Bovine Serum Albumin, Sigma) for 30 minutes; incubation with primary monoclonal antibodies (rabbit polyclonal antibodies against ADAMTS-1 amino-terminal end (Abcam 28284) and antiversican (Sigma—HPA004726) diluted in 1% PBS/BSA for a minimum of 12 hours and a maximum of 18 hours in a humid chamber at 4°C.

For the detection of primary antibodies, Alexa Fluor 488-conjugated secondary antibodies (Invitrogen, Carlsbad, CA, USA) were used. For nucleotide labeling, Hoechst 33258 (1 : 200, Sigma) was used. For better cytoskeleton visualization, we used the Alexa Fluor 568 Phalloidin (Life Technologies, Carlsbad, CA, USA). Secondary antibodies and Hoechst were diluted in PBS/BSA (Sigma) and incubated for 1 hour in a dark humid chamber at room temperature. After this, the coverslips were washed for 5 minutes with PBS solution and two times in distilled water before mounting them on glass slides using ProLong Antifade Kit (Molecular Probes). The slides were examined under a fluorescence microscope (Axio Scope.A1, Zeiss) equipped with a digital photo camera (AxioCam MRc, Zeiss).

### 2.12. Statistical Analysis

The data were analyzed using the GraphPad Prism 5 software (GraphPad Software Inc., San Diego, CA, USA). Differences between the groups were assessed by the Mann–Whitney nonparametric test. In the statistical analysis, using *p* value <0.05 was considered significant.

## 3. Results

### 3.1. RT-qPCR Showed the Expression of ADAMTS-1 and Versican in Ameloblastoma

**Figure 1:**
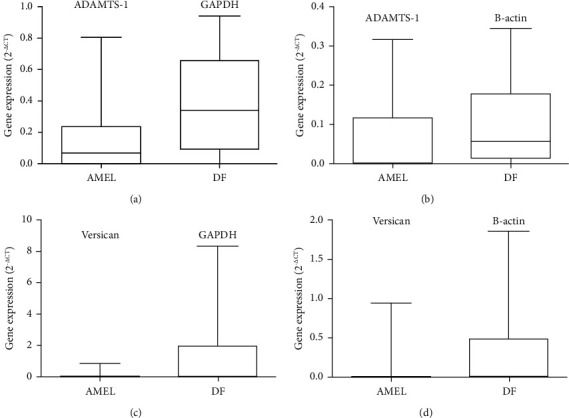
ADAMTS-1 and versican gene expression are downregulated in ameloblastoma (AMEL) when compared to dental follicle (DF). RT-PCR analysis using the Pfaffl method to calculate the relative mRNA levels normalized by GAPDH (a)–(c) or *β*-actin (b)–(d).

In our 20 samples of ameloblastoma, ADAMTS-1 had been amplified in all samples of ameloblastoma and 10 DF samples. Fifteen ameloblastoma and 6 DF samples showed expression of versican. The results of differential expression analysis between ameloblastoma and DF, a control, showed ADAMTS-1 and versican were more expressed in DF, but there was no statistically significant difference. The results were similar when using *β*-actin and GAPDH as constitutive genes (Figure 1).

### 3.2. Immunohistochemistry Analysis Showed the Highest Expression of ADAMTS-1 in Ameloblastoma

**Figure 2:**
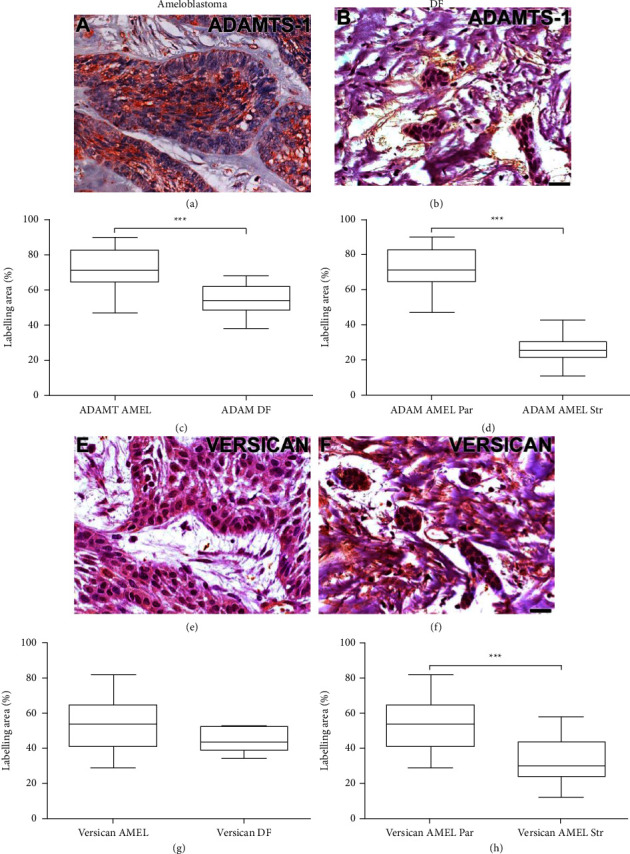
ADAMTS-1 and versican immunostaining in ameloblastoma (AMEL) and dental follicle (DF). ADAMTS-1 has stronger staining in ameloblastoma and is present in cells cytoplasm and nucleus (a) The DF also expressed ADAMTS-1, but limited to small areas. (b) ADAMTS-1 labeling area shows neoplastic cells of ameloblastoma with higher expression than epithelial cells of DF. (c) The immunostaining of ADAMTS-1 in ameloblastoma is most prominent in parenchyma (Par) than stroma (Str). (d) Versican immunoexpression in neoplastic cells of ameloblastoma in the epithelium and the stroma. (e) Dental follicle expressed versican. (f) There was no difference in labeling area of versican in ameloblastoma and DF. (g) The immunostaining of versican in ameloblastoma is most prominent in parenchyma than stroma. (h) Significance: ^*∗∗∗*^*p* < 0.001. Scale bar: 20 *μ*m.

All ameloblastoma samples showed ADAMTS-1 expression. The neoplastic cells of ameloblastoma demonstrated staining in the cytoplasm and nucleus. The DF also expressed ADAMTS-1, however, with weak labeling (Figures 2(a) and 2(b)). The neoplastic cells of ameloblastoma showed high expression than the epithelial cells of DF (Figure 2(c), *p* < 0.001). The immunostaining of ADAMTS-1 in ameloblastoma was most prominent in the parenchyma than the stroma. The value of *p* for ADAMTS-1 expression was statistically significant (Figure 2(d), *p* < 0.001).

Versican was also present in all ameloblastoma samples. There was immunostaining for neoplastic cells in the epithelium and stroma. However, the expression was more prominent in the epithelial cells of the tumor, labeling both in the nucleus and in the cytoplasm (Figure 2(e)). The dental follicle also expressed this proteoglycan (Figure 2(f)). Versican was not differentially expressed between ameloblastoma neoplastic cells and epithelial cells of DF (Figure 2(g)). The immunostaining of versican in ameloblastoma was most prominent in the parenchyma than the stroma (Figure 2(h), *p* < 0.001).

### 3.3. Western Blot Analysis Detected ADAMTS-1 and Versican Forms in AME-HPV2 Cell Lines

**Figure 3:**
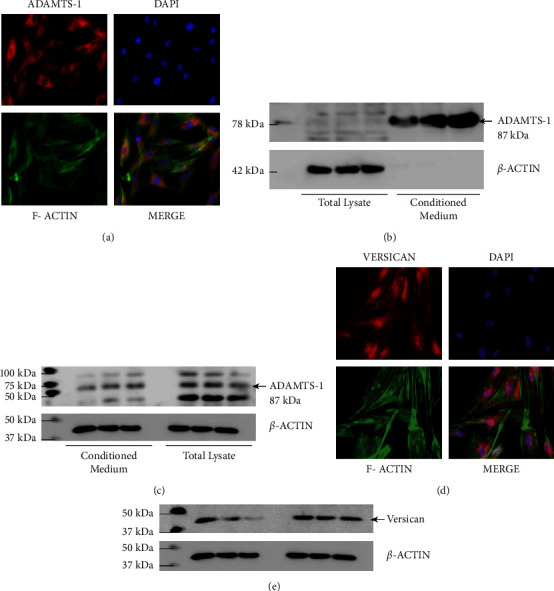
ADAMTS-1 and versican levels on AME cells line. ADAMTS-1 immunolocalization on AME cells shows cytoplasmic localization (red channel). (a) Immunoblot from cell lysate and conditioned medium. (b) Immunoblot from conditioned medium and total lysate after treatment with 2 *μ*M of monensin. (c) Loading control with *β*-actin (b). Versican immunolocalization on AME cells shows cytoplasmic and nuclear distribution (red channel). (d) Immunoblot from cell lysate and conditioned medium. (e) kDa: Kilodalton.

ADAMTS-1 full length (110 kDa) and mature (87 kDa) forms were detected in AME cell lysate (Figures 3(b) and 3(c)), showing that ADAMTS-1 activation is happening through proprotein convertase. In addition, two other bands (65 and 50 kDa) were observed in control and monensin-treated AME cells (Figure 3(c)), probably resulting from ADAMTS-1 C-terminal further processing, which impacts substrate specificity, localization of enzymes, and regulated activity. Versican fragments were detected in AME cells (Figure 3(e)), possibly cleaved by proteases as ADAMTS-1.

### 3.4. Indirect Immunofluorescence Analysis Detected ADAMTS-1 and Versican in AME-HPV2 Cell Lines

ADAMTS-1 and versican expression were detected on AME cells by indirect immunofluorescence (Figures 3(a) and 3(d)). ADAMTS-1 was observed as intense granular immunostaining (red) distributed mainly in the cytoplasm (*A*). Granular expression of versican (red) was also observed throughout the nuclei and a weak stain in the cytoplasm(*C*). Cytoskeleton is stained with phalloidin (red) and nuclei are stained with DAPI (blue), (*A* and *C*).

## 4. Discussion

Although ameloblastoma is a benign odontogenic tumor arising from the odontogenic epithelium, its behavior is characterized by being locally invasive with a high rate of recurrence [1, 27]. Cell-to-cell interactions in a complex tumoral microenvironment may be regulated by secreted growth factors and cytokines [28]. Previous studies have shown the action of enzymes of the metalloproteinases (MMP) family in ameloblastoma, showing that these enzymes are probably synthesized by tumor induction and in response to cytokines, growth factors, and hormones and are linked to tumor progression [9]. Our goal was to elucidate whether ADAMTS-1, a member of the metalloproteinase family, and versican are expressed in ameloblastoma and whether they are correlated with biological behavior in the tumor.

ADAMTS-1 is a single copy gene in the human genome, located on chromosome 21q.21.2, translating into a 110 kDa protein, and processed to a mature protein of 87 kDa. Initially, ADAMTS-1 has been described as a mediator of inflammation, but its activity has since become appreciated in organogenesis, blood/lymph vessel formation, ovarian folliculogenesis, ovulation, heart, adrenal, skeletal muscle, thyroid, stomach, and others [19, 29]. In physiological events, ADAMTS-1 remodels the ECM through the proteolytic degradation of substrates such as chondroitin sulfate proteoglycans. The dysregulation of ADAMTS-1 often induces to pathological manifestations of altered ECM and/or vascular density, and many studies have highlighted its functional activity during tumorigenic transformation [18, 21, 30].

This is the first time that a member of the ADAMTS family has been described with ameloblastoma. In the present study, all samples of ameloblastoma expressed the ADAMTS-1 mRNA by RT-PCR and immunohistochemical analysis showed high expression in protein. There was strong staining of this protein in the center of epithelial islands dispersed in the connective tissue. Although ADAMTS-1 has been expressed more in DF than in ameloblastoma by RT-PCR, the immunochemistry analysis showed high ADAMTS-1 expression in ameloblastoma. Expression differences between techniques are common and can be explained by molecule preservation, or a possible explanation might be that mRNA levels are a reflection of the average gene expression in the entire FFPE slice, whereas IHC may be biased in favor of representative areas. We can also consider posttranslational modifications, such as miRNA interference, in DF samples that could be contributing to the fact that the ADAMTS-1 protein is not so high. miRNA 181-d induced downregulation of ADAMTS-1 in adipose tissues [31] and this miRNA was present in saliva fluid associated with developing and erupting teeth [32]. Therefore, miRNA 181-d may downregulate the translation of ADAMTS-1 in DF. In the Western blot analysis on AME cells, ADAMTS-1 full-length (110 kDa) and mature (87 kDa) forms were detected by immunoblot on AME cell lysate. In addition, two other bands (65 and 50 kDa) were observed in control and monensin-treated AME cells. The lighter bands were initially considered as in specific but can be a result of ADAMTS-1 *C*-terminal further processing [16, 33]. Our previous work also showed ADAMTS-1 in breast cancer cells with different processing status [20], and indirect immunofluorescence detected ADAMTS-1 expression in AME cells.

The mature form (87 kDa) and the truncated active form (65 kDa) of ADAMTS-1 were tested by cleavage substrate, the lower form was rather inefficient at processing otherwise the mature form was more efficient at cleaving. The *C*-terminally processed 65 kDa form lacks a part of the spacer region and two *C*-terminal TS repeats. The loss of part of the ancillary *C*-terminal domains of the truncated form of ADAMTS-1 has an impact on substrate specificity, localization of enzymes, and regulated activity [12, 16, 34].

Furin plays an important role in the maturational processing of ADAMTS-1. Furin is a proprotein convertase, which cleaves ADAMTS enzymes at the consensus motif R/KXnR/K [13, 15]. Most often, proprotein cleavage by furin occurs in the trans-Golgi network and shuttles to the cell surface and back via endosomes. However, furin has also been shown to operate extracellularly, in a shed, soluble form [16]. In our work, for the Western blot analysis at the first moment, there were no detected proteins in the cell lysate, only detected in the supernatant. Then, monensin was used and increased the ADAMTS-1 content in the cytoplasm since the protein secretion is impaired with Golgi complex disruption [35].

In addition, it has been reported that dental pulp cells, odontoblasts, cementoblasts, cementocytes, osteoblasts, osteocytes, and periodontal ligament cells regulate the temporal and spatial expressions of the extracellular accumulation of versican using the degrading enzymes, ADAMTS-1, 4, and 5, which suggests cooperation of these molecules in remodeling the extracellular environment surrounding these cellular phenotypes at the tooth eruption [36]. During the pathological process, ADAMTS-1 can cleave or induce the release of proangiogenic factors (IGFBP2, HB-EGF, AR) and degrade ECM components, such as versican, to facilitate tumor invasion. Protumor effects are protease-dependent. In addition, this protein can induce the recruitment of fibroblasts involved in tumor growth. Alteration of the tumor microenvironment is essential to promote tumor growth and invasion. The activities of this protease have been associated with an increase in the tumorigenic potential of neoplastic cells. ADAMTS-1 can be expressed by tumor cells or tumor stromal cells and contributes to modifications in the tumor microenvironment by proteolytic or independent activity-dependent mechanisms [14, 37].

Besides, ADAMTS-1 was found to be significantly increased in the oral environment in chronic and aggressive periodontitis patients. In this search, there was a correlation between ADAMTS-1, hypoxia-inducible factor-1alpha (HIF-1*α*), and vascular endothelial growth factor (VEGF-A) in gingival crevicular fluid patients and this correlation could play a role in the pathogenesis of the disease [38]. It has been reported that hypoxia also has been linked to increased expression of ADAMTS-1 in renal fibrosis tissues [39]. Our group showed an interesting pattern of HIF-1*α* immunostaining in ameloblastoma [40]. Possible hypoxia conditions are involved in the expression of ADAMTS-1 in ameloblastoma.

Furthermore, ADAMTS-1 and MMP-1 act in concert to not only enhance invasion through the ECM and endothelium but also to promote tumor colonization in the bone microenvironment through an intricate pro-osteolytic signaling cascade that involves tumor cells, osteoblasts, and osteoclasts. MMP-1 and ADAMTS-1 proteolytically release EGF-like ligands, including amphiregulin (AREG), heparin-binding EGF (HB-EGF), and transforming growth factor *α* (TGF *α*), from tumor cells. These EGF family growth factors signal through the EGFR pathway in osteoblasts to inhibit the expression of osteoprotegerin (OPG). OPG, a soluble decoy receptor of receptor activator of nuclear kB ligand (RANKL), is also produced by osteoblasts to antagonize the activity of RANKL, which is expressed in both membrane-bound and soluble forms by osteoblasts, while its cognate receptor RANK is expressed on the surface of osteoclasts and controls a key signaling pathway essential for osteoclast differentiation. Furthermore, increased MMP-1 and ADAMTS-1 expressions are associated with an increased risk of bone metastasis in breast cancer patients [41]. As in this study, ameloblastoma also expressed MMP-1 [42] and ADAMTS-1, which were highly expressed in our study, suggesting that ADAMTS-1 and MMP-1 could have the same mechanism and may be associated with the behavior of ameloblastoma.

Based on this finding and the high expression of ADAMTS-1, protein in the ameloblastoma in the present study may suggest that this enzyme may participate in the tumor invasion mechanism of this neoplasia, especially for the degradation of the substrate (versican) in the ECM.

Versican is synthesized by a variety of cells and in humans, it is encoded by chromosome 5q14.3. This gene has 86% homology between mice and humans, indicating its importance and highly conserved nature. Versican is a large chondroitin sulfate/dermatan sulfate proteoglycan in the ECM. Its core protein consists of two globular domains, *G*1 and *G*3 at the *N* and *C*-terminal, respectively, and up to two CS-attachment domains. Versican has four isoforms: *V*0 contains both CS*-α* and CS-*β* domains, *V*1 contains CS-*β* domains, *V*2 contains CS-*α*, and *V*3 contains neither of them [43, 44]. *V*0 and *V*1 are present in various tissues in the body, such as the cardiovascular system, muscle tissue, embryonic tissue, and others. *V*2 is expressed mainly in the brain tissues of adults. *V*3 has a low expression in adult tissues [45].

Versican expression is associated with a proliferative cell phenotype and is often found in tissues that show high proliferation, such as embryonic tissues, and in a variety of tumors, including breast, brain, prostate, and melanoma [46]. In the pathological process, when versican is cleveated, the *G*1 domain stimulates proliferation by destabilizing cell adhesion, whereas the *G*3 domain induces proliferation, at least in part, by activating EGFR via the action of epidermal growth factor (EGF) like motifs. *G*1 and *G*3 domains may differentially control tumor growth rate and have interactive roles to promote tumor development and metastasis. Notably, several protease families that include ADAMTS, MMPs, and plasmin can cleave versican generating fragments containing the *G*1 domain in some cases. Thus, the regulation of *G*1 and *G*3 versican levels by proteases is an important factor in cancer cell motility and metastasis [5, 47]. Our previous work showed that ADAMTS-1, versican, and pEGFR were expressed in chronic periapical lesions and this protein interaction may participate in the pathogenesis of granuloma and radicular cysts, through the remodeling of the ECM (versican) by ADAMTS-1, producing bioactive fragments, which could activate EGFR, contributing to the formation, growth, and maintenance of injuries [48]. We hypothesized the same mechanism that may occur in ameloblastoma.

Furthermore, it was found strong immunoreactivity in the epithelium of versican in the peripheral region of salivary pleomorphic adenoma [49]. High versican immunoexpression was observed in all kinds of odontogenic myxoma [50]. While other findings observed that there was a positive versican expression in the stroma of odontogenic tumors [51].

In the present study, 16 samples of ameloblastoma expressed versican genes, only 4 samples were not expressed by RT-PCR, but all samples of ameloblastoma expressed versican by immunochemistry. 6 samples of DF were expressed versican, 4 samples were not expressed by RT-PCR, and all samples of DF expressed versican in immunochemistry. The versican expression was higher in DF than in ameloblastoma by RT-PCR; however, there was a similar pattern between ameloblastoma and DF in immunochemistry analysis. We can also consider posttranslational modifications, such as miRNA interference, in dental follicle samples that contribute to the fact that the versican protein is not so high. miRNA 143 induced downregulation of versican in smooth muscle cells [52] and this miRNA was associated with tooth development [53]. Therefore, miRNA 143 may downregulate versican in dental follicles. In Western blot analysis on AME cells at the first moment, there were no detected proteins in the cell lysate, only detected in the supernatant. Then, the use of monensin was proposed since the protein could be excreted rapidly by the Golgi complex in the cell lines and a fragment of versican was observed, which possibly was cleaved by ADAMTS-1. Moreover, indirect immunofluorescence detected versican expression in AME cells.

Furthermore, in dental tissues, the expression of versican or related large proteoglycans (PGs) has been mainly observed in mesenchymal tissues such as dental papillae, dental pulp in bovines and rats, and periodontium in rats. For the dental epithelium, it has been reported versican mRNA expression in the outer enamel epithelium at the early bell stage of mouse tooth germ [36, 54]. It has been reported the immunohistochemical localization of antibody 5D5, which recognizes the core protein of large PGs, in secretary ameloblasts at the late bell stage of human tooth germ and porcine gingival epithelium, respectively [55]. Jiang et al. [56] were the first to confirm that dental epithelium can synthesize significant amounts of versican, suggesting that versican could have some role in the tumorigenesis of ameloblastoma, which in this study was expressed, given that the origin of this tumor is also the odontogenic epithelium still undifferentiated.

The methods used for the development of this study (IHC, WB, PCR) are limited, and the results and conclusions need confirmation, with studies that investigate the biological mechanisms involved with the findings. In addition, studies with more expressive samples of ameloblastoma should be performed to confirm the results.

## 5. Conclusion

The present study demonstrated an expression of ADAMTS-1 mRNA in ameloblastoma, DF by RT-PCR, and immunostaining, which showed ADAMTS-1 protein was highly expressed in ameloblastoma than in DF. In addition, Western blot and indirect immunofluorescence analysis on AME cells showed the expression of ADAMTS-1 and versican. Versican has been detected as a fragment by Western blot, possibly cleaved by proteases. These results suggest that the ADAMTS-1 protein may have any participation in the biological behavior of ameloblastoma, which is locally invasive, through the degradation of its substrate (versican).

## Data Availability

The data used to support the findings of this study are available from the corresponding author upon request.

## Abbreviations

ADAMTS: A disintegrin and metalloproteinase with thrombospondin motifs
AREG: Amphiregulin
BSA: Bovine serum albumin
COMP: Cartilage oligomeric matrix protein
DAB: Diaminobenzidine
DF: Dental follicle
ECM: Extracellular matrix
EGF: Epidermal growth factor
EGFR: Receptor EGF
FFPE: Formalin fixation paraffin embedding
HB-EGF: Heparin-binding EGF
PBS: Phosphate-buffered saline
RT-PCR: Real-time polymerase chain reaction
TGF *α*: Transforming growth factor *α*
TSR: Thrombospondin
VEGF-A: Vascular endothelial growth factor.
